# Interaction of MSC with tumor cells

**DOI:** 10.1186/s12964-016-0143-0

**Published:** 2016-09-08

**Authors:** Catharina Melzer, Yuanyuan Yang, Ralf Hass

**Affiliations:** 1 Biochemistry and Tumor Biology Lab, Department of Obstetrics and Gynecology, Hannover Medical School, Carl-Neuberg-Str. 1, D, 30625 Hannover, Germany; 2 Tongji Hospital Affiliated Tongji University, Shanghai, China

**Keywords:** MSC, Mesenchymal stroma/stem cells, Tumor cell signaling, Tumor microenvironment, Cellular interaction, Cell fusion

## Abstract

Tumor development and tumor progression is not only determined by the corresponding tumor cells but also by the tumor microenvironment. This includes an orchestrated network of interacting cell types (e.g. immune cells, endothelial cells, fibroblasts, and mesenchymal stroma/stem cells (MSC)) via the extracellular matrix and soluble factors such as cytokines, chemokines, growth factors and various metabolites. Cell populations of the tumor microenvironment can interact directly and indirectly with cancer cells by mutually altering properties and functions of the involved partners. Particularly, mesenchymal stroma/stem cells (MSC) play an important role during carcinogenesis exhibiting different types of intercellular communication. Accordingly, this work focusses on diverse mechanisms of interaction between MSC and cancer cells. Moreover, some functional changes and consequences for both cell types are summarized which can eventually result in the establishment of a carcinoma stem cell niche (CSCN) or the generation of new tumor cell populations by MSC-tumor cell fusion.

## Background

### Tumor microenvironment and associated cell populations

Solid tumors can be regarded as a complex organ with tumor cells and a variety of differentially organized cell types, establishing a certain immune status, contributing to blood vessel formation and neovascularization, and building an extracellular matrix which enables the associated cell populations to communicate within this tumor microenvironment (TME) [1, 2]. For further insights in tumor development and chemotherapeutic approaches, it is indispensable to understand the interplay of specific components of the TME, the occurring cellular communication processes and the resulting functions of this network between cancer cells and the various tumor-associated cell populations.

Predominant cell types within the TME are represented by immune cells, fibroblasts, pericytes, endothelial cells, mesenchymal stroma/stem cells and sometimes adipocytes [3]. Immune cells present in the TME involve cells from both, the innate and adaptive immune system whereby lymphocytes represent the majority of tumor-infiltrating immune cells [4]. It is not surprising that immune responses inhibit tumor development but recent studies have also proposed that immune cells can promote cancer growth. CD8^+^ T cells and CD4 T_H_1 T cells mainly exhibit anti-cancer effects since strong infiltration of tumor tissue by these cell types is associated with favorable prognosis in many solid tumors including breast, ovarian, cervical, lung and colorectal cancer [5]. In contrast, other T lymphocyte populations such as T_H_2 and T_reg_ cells have been correlated with poor clinical outcome in several cancer types [5]. Likewise, B lymphocytes are attributed a dual role in tumorigenesis. Whereas high numbers of B cells in the tumor stroma are linked to favorable prognosis in breast cancer, mouse models revealed opposite results assigning a tumor promoting role for B lymphocytes [6, 7]. Moreover, natural killer and natural killer T cells also found in the TME are proposed to support favorable clinical outcome [3, 8]. Tumor-associated macrophages and myeloid suppressive cells represent tumor-promoting immune cells together with their derived cytokines IL-6, IL1β, IL23 and TNFα [9]. For instance, tumor-associated macrophages can interact with metastasizing breast cancer cells in the lung via VCAM-1 and promote tumor cell survival. Furthermore, released pro-inflammatory cytokines such as TNFα contribute to increased migration and invasiveness of breast and ovarian cancer cells [10].

In perivascular niches of tumor blood vessels pericytes are predominantly present and can associate with vascular endothelial cells by contributing to the formation of blood capillaries [11]. In contrast to normal healthy vasculature, tumor vessels display an abnormal physiology due to aberrant pericyte coverage and leaky endothelial layers upon increasing hypoxia [11]. Thus, normalization of tumor vasculature to enhance drug delivery and to reduce hypoxia in the tumor stroma might be a promising therapeutic approach since low pericyte coverage is associated with poor prognosis and pericyte depletion has been correlated with enhanced hypoxia and metastasis [12].

Further cell populations are represented by stromal cells or fibroblasts within the TME. The so-called cancer- or carcinoma-associated fibroblasts (CAFs) which are derived from various precursors like aberrant mesenchymal stroma/stem cells (MSC) or endothelial cells secrete a plethora of growth factors, cytokines, chemokines, structural protein components, and metabolites that communicate with tumor cells and promote oncogenesis by activating cell proliferation, tumor angiogenesis and invasive properties [3]. Thus, aberrant tumor-associated MSC can acquire different functions following interaction with tumor cells including enhanced secretion of TGF-β to contribute to epithelial-to-mesenchymal transition (EMT) and immune-suppressive activities. Moreover, these aberrant MSC release VEGF for neo-vascularization within the TME and they produce CXCL12 (=SDF1 (stromal cell-derived factor 1)) to support tumor cell growth and survival [3].

Likewise, MSC are one of the key players within the TME and can either inhibit or promote tumor cell growth by distinct types of cellular interaction [13]. Reduction of tumor growth by MSC can be mediated via inhibited angiogenesis, suppressed Wnt and AKT signaling, or induction of cell cycle arrest and apoptosis [14, 15].

MSC are recruited to tumor sites and can be activated by certain stimuli such as TGF-β1 to develop a CAF-like phenotype [16].

In addition to a variety of different cell populations in the tumor microenvironment, the extracellular matrix (ECM) also plays an important role in the regulation of tumor development und progression. The ECM does not only provide a structural scaffold for the tumor stroma with fibrous proteins such as elastin, collagen and fibronectin, and proteoglycans like chondroitin sulfate and hyaluronic acid, but in addition, ECM is abundant source of soluble factors including growth factors, angiogenic factors, cytokines and chemokines. This dynamic and complex network contributes to the intercellular cross-talk with cancer cells. During tumor development the ECM is usually dysregulated, remodeled and appears disorganized [17, 18]. Collagens are the most abundant fibrous proteins in the extracellular matrix. Nevertheless, collagen deposition and cross-linking or tight association with other structural matrix proteins such as elastins, laminins or fibronectin has been associated with cancer invasion and metastasis [19]. Cross-linking of collagen by modifying enzymes such as lysyl oxidases leads to a more rigid phenotype of the whole tumor [20]. Stiffness of the tumor stroma causes intracellular contraction and a more rigid cytoskeleton which in turn leads to a higher migratory capacity [17].

Taken together, the tumor microenvironment contains a variety of initially non-malignant cell types (immune cells, endothelial cells, fibroblasts, MSC) which develop tumor-associated functionalities together with soluble factors and ECM components that all communicate with cancer cells thereby inhibiting and promoting tumorigenesis. Nonetheless, it is important to strengthen that the TME is a dynamic and heterogeneous environment whose total composition varies between tumors and patients. However, the tumor stroma exhibits common features of these distinct cell types which may serve as interesting therapeutic targets [18].

### Role of mesenchymal stroma/stem cells and possible interactions

MSC are multipotent cells that preferentially reside in perivascular niches of nearly all human tissues and organs like bone marrow, adipose tissue, heart or lung and neonatal tissues including placenta, amniotic membranes or umbilical cord [21–24].

Apart from various sources and heterogeneous populations, MSC exhibit certain common properties ranging from the expression of surface markers (CD73, CD90, CD105) to the differentiation along the adipogenic, chondrogenic and osteogenic lineage [25]. Their functions are extremely diverse and depend on the tissue-specific origins and the special microenvironment in which MSC are embedded. Accordingly, in vitro cultured MSC can develop different morphologies and properties whereby long-term stemness can be maintained which includes MSC cultures for up to 10 passages without loss of proliferative capacity, telomerase activity or differentiation capacity [26–28].

In addition to the MSC heterogeneity, subpopulations are characterized with altered proliferative capacity and aging properties [29] which may also include epigenetic changes. Selective MSC subtypes carry additional surface markers such as Stro-1 [30], CD146 [31], the chemokine receptors VCAM-1 (CD106) and ICAM-1 (CD54) [32] predominantly found in bone marrow-derived MSC, CD271 [33], or the more embryonic like stem cell markers Oct-4 and Sox2 [34], which accompany the multi-facetted MSC functionalities and affect interactions with other cell types.

MSC are recruited to sites of injury to support tissue repair, stem cell homeostasis and immunomodulation. Similar functions are displayed by MSC during tumor development, whereby permanently proliferating and invasively growing tumor cells create an inflammatory microenvironment displaying a certain kind of “wound that never heals” [35]. Thereby, MSC exhibit tissue repair functions and support angiogenesis which simultaneously contributes to promote the growth of cancer cells [35, 36]. Migration of MSC towards the inflammation site leads to cellular interactions that occur both directly via gap junctions, membrane receptors and nanotubes and indirectly via soluble structures and factors. Through releasing of different endocrine and paracrine signals, MSC stimulate neighbored cells with pro- and/or anti-tumorigenic activities. In turn, MSC can be stimulated by tumor cells to develop an aberrant tumor-associated phenotype [14].

### Direct and indirect interaction of MSC with tumor cells

**Fig. 1:**
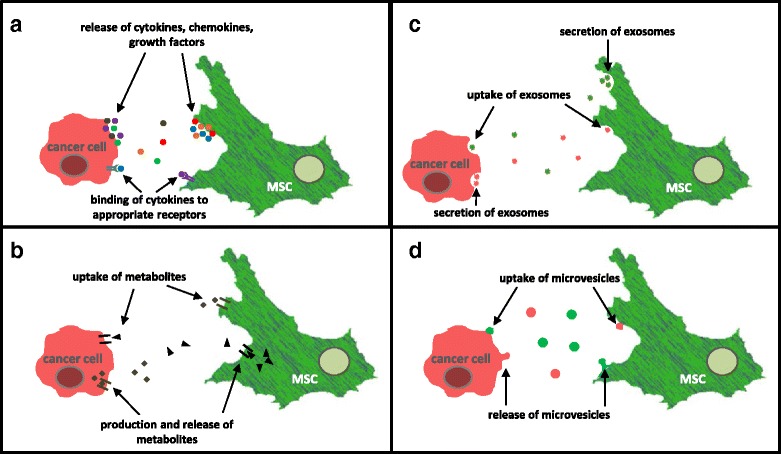
Indirect interactions between mesenchymal stroma/stem cells and cancer cells. **a** Cytokines, chemokines, growth factors: MSC secrete a plethora of soluble factors that can bind as substrates to appropriate receptors on the cell surface of cancer cells and vice versa for mutual activation of signaling pathways. **b** Metabolites: Likewise, MSC-released metabolites such as prostaglandin E2, kynurenine or galectin-1 can act in a paracrine manner on cancer cells altering their properties and functions [14]. **c** Exosomes: Both, MSC and cancer cells, secrete exosomes for the exchange of small molecules including protein, mRNAs and microRNAs. **d** Microvesicles: Besides exosomes, microvesicles represent a different type of microparticles for the exchange of small molecules such as mRNAs or microRNAs affecting tumor cells and MSC in mutual ways

**Fig. 2:**
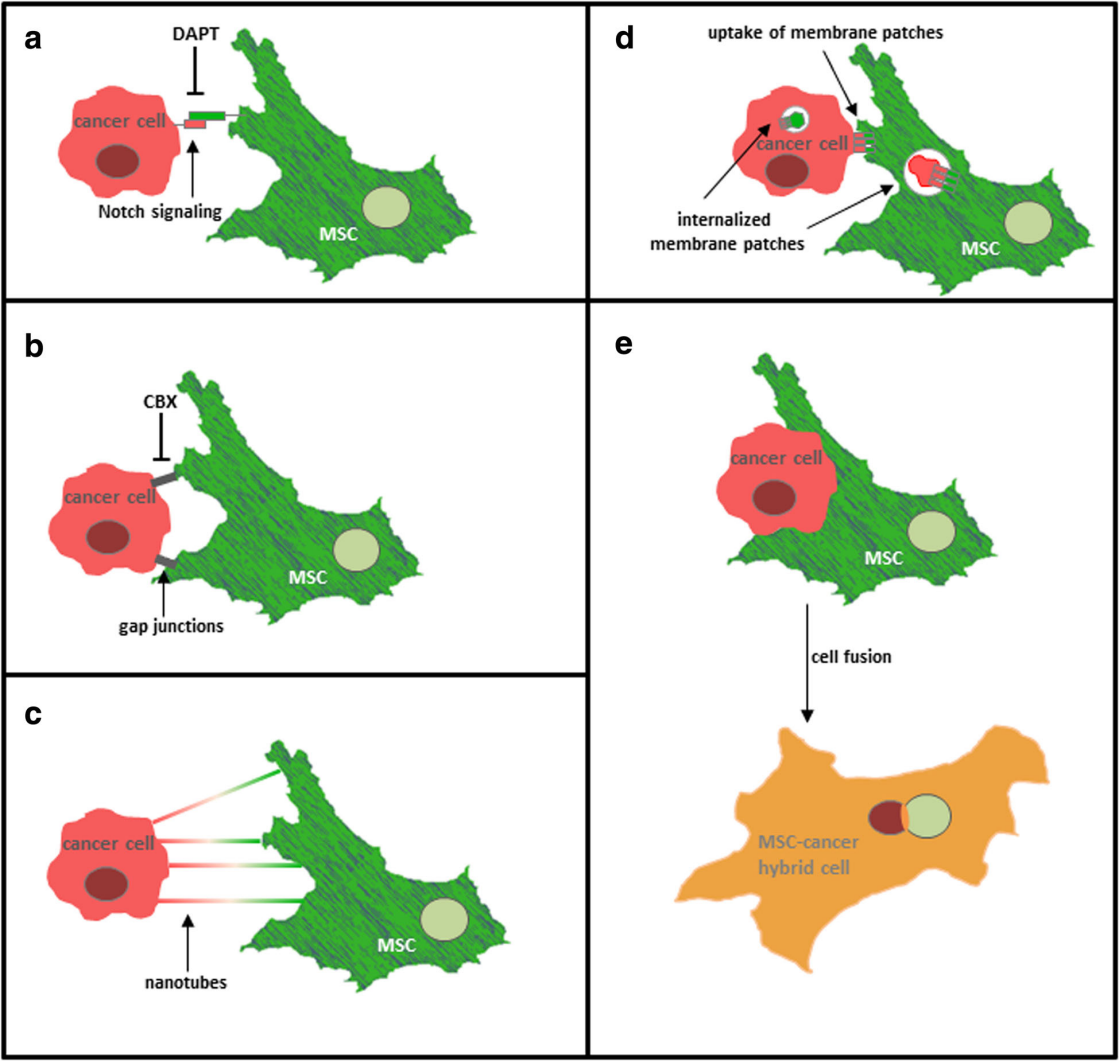
Direct interactions between mesenchymal stroma/stem cells and cancer cells. **a** Notch signaling: A prominent example for direct cell-to-cell interaction is represented by Notch signaling. DAPT, a Notch signaling inhibitor, was shown to decrease functional alterations of breast cancer cells after co-culture with MSC underlining the involvement of Notch signaling in MSC-cancer cell interactions. **b** GJIC: Both MSC and cancer cells build gap junctions for intercellular communication. Gap junctional intercellular communication (GJIC) can be inhibited by gap junction inhibitor carbenoxolone (CBX) resulting in lesser interactions and functional alterations of cancer cells and MSC. **c** Nanotube formation: Long and thin plasma membrane structures formed between MSC and cancer cells allow the transport of small molecules and organelles for cellular cross-talk leading to altered functions and phenotype. **d** Trogocytosis: An exchange of whole plasma membrane fragments via an active transfer outlines a further possible interaction type between MSC and cancer cells resulting in structural and functional alterations of the recipient cell. **e** Cell fusion: In rare cases, mesenchymal stem cells have the capacity to fuse with various cancer cell types such as breast, ovarian, lung and gastric cancer cells. The molecular mechanism about the formation of such cancer hybrid cells is still unknown

Different types of cross-talk between MSC and cancer cells both directly and indirectly are illustrated in Figs. 1 and 2, respectively. Several direct and/or indirect mechanisms of interaction contribute to MSC-mediated stimulation of cancer cell growth including Notch signaling, nanotube formation, gap junctional intercellular communication, and/or the exchange of cytokines/chemokines, extracellular vesicles and exosomes [36–38]. It is thus important to emphasize that these different types of indirect and direct interactions are always multidirectional, therefore affecting and altering both, the tumor cells as well as the MSC or other cellular partners.

### Indirect interaction of MSC with tumor cells

#### Cytokines, chemokines and growth factors

Indirect communication proceeds via secretion of soluble molecules (e.g. growth factors, cytokines, and chemokines) that might function as substrates for specific receptors of neighboring cells to activate intracellular signaling pathways. Activated MSC produce and secrete a large variety of bioactive molecules suggesting MSC as Medicinal Signaling Cells [39]. MSC-mediated release of these biological compounds can affect adjacent populations like tumor cells as cellular modulators. For instance, previous studies revealed that secretion of the CC-chemokine ligand 5 (CCL5) by MSC also known as RANTES (regulated on activation, normal T cell expressed and secreted) can interact with appropriate chemotactic cytokine receptors such as CCR1, CCR3 or CCR5 [35]. Moreover, CCL5 can activate the G-protein coupled receptor GPR75 in breast cancer cells in a paracrine manner. Such CCL5 signaling among further stimuli resulted in acceleration of migratory, invasive and metastatic capacity of the breast cancer cells [35] (Fig. 1).

#### Metabolites

Various metabolites within the TME released and exchanged by the different interacting cell populations strongly affect the progress of malignancy and contribute to alter tumor cell properties such as motility, survival and self-renewal. These effects are relayed in part by altered energy turnover and consumption whereby lactate, glutamine and keton bodies contribute to a functional change of cancer cells toward an OXPHOS addicted phenotype [40].

Besides the secretion of cytokines and chemokines into the tumor stroma, several metabolites such as prostaglandins or indoleamine 2,3-dioxygenase (IDO) represent further released soluble factors stimulating cells in a paracrine manner and contributing to the interaction network of the TME [41, 42].

Previous studies observed that cyclic adenosine monophosphate (cAMP) can inhibit DNA damage-induced p53 accumulation via activation of protein kinase A (PKA) resulting in increased survival of lymphoblastic leukemia cells [43, 44]. In this context, MSC-derived prostaglandin E2 contributed to protect lymphoblastic leukemia cells from DNA-damage induced p53 accumulation and cell death via PKA supporting a tumor promoting role of MSC in the TME [45, 46].

A close metabolic coupling of MSC was demonstrated with osteosarcoma cells whereby tumor cell-induced oxidative stress in MSC was associated with higher levels of lactate and lactate efflux receptors. Consequently, osteosarcoma cells increased expression of lactate influx receptors whereby lactate secreted by MSC and incorporated by osteosarcoma cells elevated ATP production and increased migratory capacity of the cancer cells [47]. In general, metabolic modifications within the TME including osmolarity, hypoxia or acidification influence tumor cell growth and appropriate malignancy [48].

#### Microparticles

Another indirect interplay between MSC and cancer cells is represented by the exchange of microparticles including exosomes and microvesicles. Whereas exosomes are defined as small homogeneous membrane particles of endocytic origin ranging in size from 40 to 100 nm, microvesicles are directly shed from the plasma membrane into the extracellular environment representing a larger and heterogeneous population with 50 to 1000 nm in diameter [49]. Although both types of microparticles differ in size, origin and releasing mechanism, exosomes and microvesicles contain a large panel of proteins, functional mRNAs and regulatory microRNAs (miRs) which contribute to the cellular interplay between MSC and cancer cells within the tumor microenvironment and thereby altering the functionality of recipient cells [37].

Previous results demonstrated that MSC-derived exosomes can modulate the function of tumor cells by induction of MMP-2 and ecto-5’-nucleotidase activity resulting in a more complex tumor microenvironment with higher tumor heterogeneity [37, 50]. Alternatively, MSC-derived exosomes also contain tumor supportive miRs which enhance tumor growth in vivo [51]. Vice versa, cancer cells secrete exosomes as well and recent work demonstrated that prostate cancer cell-derived exosomes stimulate the differentiation of bone marrow-derived MSC into pro-angiogenic myofibroblasts with tumor growth promoting functions [52].

Anti-tumor effects have been also observed with microvesicles derived from human umbilical cord Wharton’s jelly MSC which inhibit bladder tumor cell growth via cell cycle arrest and induction of apoptosis, both in vitro and in vivo [53].

Although functional mechanisms for these controversial observations of tumor promoting versus tumor inhibiting roles of MSC-derived microvesicles and exosomes remain to be elucidated, it appears conceivable that the cargo of these microparticles which depends on the activation status and state of development of the originating MSC is primarily responsible for the type of action on tumor cells.

### Direct interaction of MSC with tumor cells

#### Notch signaling

Notch signaling plays an important role in fundamental processes such as support of tissue repair or regulation of various immune cell functions [54]. Activation of notch signaling involves ligand binding to the notch receptor, cleavage of the intracellular domain of the notch receptor by a presenilin-γ-secretase and translocation of this cleavage domain into the nucleus thereby resulting in trans-activation of downstream target genes [55] (Fig. 2).

Previous experiments indicated a functional involvement of the notch pathway during the interaction between MSC and breast cancer cells [36]. Inhibition of notch signaling via N-[N-(3,5-difluorophenacetyl-lalanyl)]-S-phenylglycine t-butyl ester (DAPT), a γ-secretase inhibitor, reduces MSC-mediated CD90 expression and growth of breast cancer cells in co-culture experiments [36, 56]. Moreover, DAPT could partially reduce MSC-induced EMT in pancreatic cancer cells emphasizing the involvement of notch signaling during MSC-cancer cell interactions [57].

#### Gap junctional intercellular communication (GIJC)

Gap junctions connect adjacent cells for intercellular, direct communication called gap junctional intercellular communication (GJIC) which can regulate cell growth and differentiation or maintain tissue homeostasis. One gap junction channel is composed of two hemi-channels from each interacting cell. One hemi-channel is formed by 6 connexin protein subunits and each connexin in turn features four transmembrane domains. Typically small molecules and second messenger such as cAMP and Ca^2+^-ions are transported through gap junctions [58].

Acquisition of CD90 by breast cancer cells after co-culture with MSC is associated with GJIC signaling since carbenoxolone, a gap junction inhibitor, reduces MSC-mediated CD90 expression of breast cancer cells [36].

#### Nanotubes

Nanotubes represent thin, dynamic cytoplasmic protrusions which connect two cells enabling the exchange of a variety of biological cargo ranging from organelles such as mitochondria to small molecules including calcium ions and glycoproteins over longer distances [59].

Besides exchange of cargo between tumor cells themselves [60], cancer cells have been reported to form nanotubes with MSC as well [61]. In particular, breast cancer cells MDA-MB-231 acquire mitochondria from bone-marrow derived MSC via nanotubes resulting in altered metabolic activity and increased proliferative and invasive capacity [61].

#### Trogocytosis

A further mechanism for direct cross-talk between MSC and cancer cells is displayed by trogocytosis. This type of direct cellular interaction was firstly described between immune cells for the transfer of surface molecules from antigen-presenting cells to lymphocytes as an active mechanism [62]. Likewise, trogocytosis has been observed between MSC and cancer cells. Thus, ovarian cancer cells captured stromal membrane patches resulting in chemoresistance [63]. Moreover, trogocytosis has been suggested during interaction of MSC with a variety of tumor cells including ovarian cancer and breast cancer cells [38].

#### Cell fusion

The closest/strongest and most complex interaction between MSC and cancer cells is the formation of fusion or hybrid cells which also represents a very rare event whereby underlying molecular mechanisms are still not fully understood. Co-culture of MSC with cancer cells like breast or ovarian cancer at certain conditions in vitro can lead to the development of hybrid cells by fusion of the two parental cell lines [38]. Potential fusion events depend on the cell density, the cell ratio of the parental populations, the medium components and culture conditions (ionic strength, pH, hypoxia) among others. However, the associated signaling pathways and the precise requirements either favoring or inhibiting such hybrid cell formations remain unclear. Intercellular fusion in general represents an intricate and highly regulated event which plays an important role in fundamental processes during development, for instance during fertilization between sperm and egg [64]. Although various studies suggest a contribution of tumor cell hybrids to cancer metastasis, there is still little known about cell fusion in pathophysiological processes like cancer and the role of cell-fusion tumor products.

**Fig. 3:**
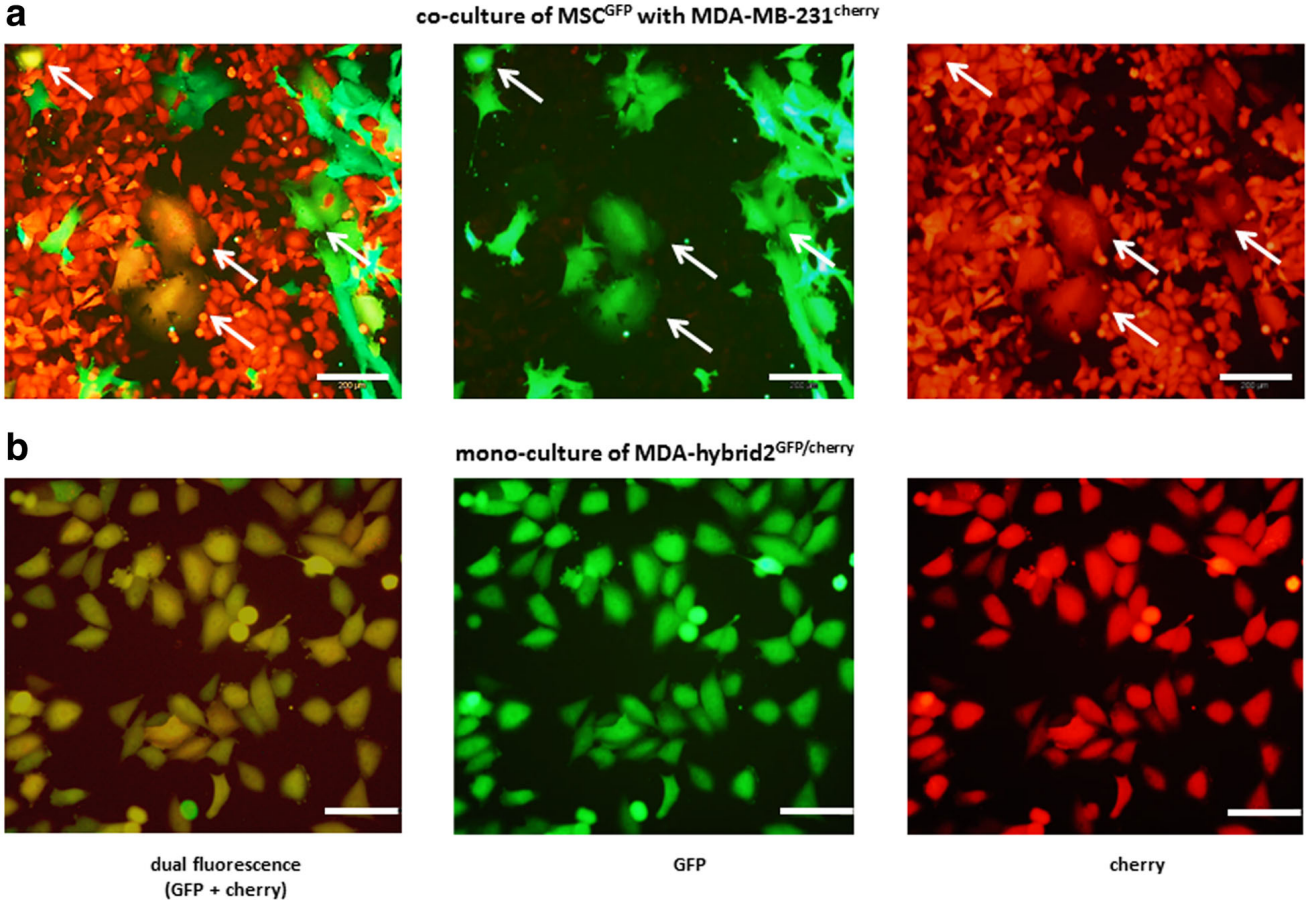
Formation and culture of tumor hybrid cells after spontaneous cell fusion. **a** Co-culture of MSC^GFP^ with MDA-MB-231^cherry^ breast cancer cells demonstrating the development of fusion cells which are indicated by *white arrows*. Scale bars represent 200 μM. **b** Mono-culture of isolated and expanded MDA-hybrid2 cells exhibiting dual fluorescence from both maternal cell populations (MSC^GFP^ and MDA-MB-231^cherry^). Scale bars represent 100 μM

Nevertheless, hybrid cells from human breast cancer and breast epithelial cells are well characterized [65] and spontaneously fused hybrid cells have been reported in several co-cultures between MSC and various cancer cell lines including breast, ovarian, lung and gastric cancer [36, 38, 66–68]. Figure 3a exemplarily outlines a co-culture of primary human umbilical cord-derived MSC and the breast cancer cell line MDA-MB-231. In order to distinguish both populations, MSC and tumor cells were stably transduced with a lentiviral vector carrying either the eGFP or the mCherry gene, respectively, resulting in green-fluorescing MSC^GFP^ and red-fluorescing MDA-MB-231^cherry^. In such a co-culture system with MSC and cancer cells, yellow-fluorescing hybrid cells are spontaneously formed within 3 to 6 days whereby the cell size and shape varies depending on the culture conditions and the contribution of parental cell populations [36].

Besides the labeling of both parental cell lines with fluorescent lentiviral vectors, another system has been applied to clearly identify hybrid cells in a co-culture. The so-called bimolecular fluorescence complementation involves a lentiviral transduction of both parental cells as well whereby the eGFP gene is separated into two non-fluorescent halves and each cell type is transfected with one of these halves. Consequently, only a fusion between a mesenchymal stem cell and a cancer cell can bring these two different halves together resulting in a functional GFP fluorescence after expression that is detectable via fluorescence microscopy or flow cytometry [66].

Fused hybrid cells generated in a labeled co-culture can be isolated by FACS and subsequent single cell cloning [68]. A successful isolation and expansion of one single cell clone derived from a co-culture between MSC^GFP^ and MDA-MB-231^cherry^ is displayed in Fig. 3b.

Whereas knowledge about tumor cell fusion remains scarce, certain characteristics of properties and functions are available about MSC-cancer hybrid cells.

Formation of hybrid populations was reported in various studies between MSC and breast cancer as well as ovarian cancer cells [14, 36, 38]. Moreover, in vitro fusion was documented in MSC which were derived from human embryonic stem cells and fused with various breast cancer cell lines including MDA-MB-231, T47D, MCF7 and MCF10A. These hybrids acquired characteristics from both parental cell types (MSC and breast cancer cell) such as enhanced migratory capacity and expressed high motility like MSC, but their migration movement was nondirectional similar to the breast cancer cells [66]. Likewise, a higher migration rate and concomitant acquisition of distinct MSC-like characteristics was assigned to breast cancer hybrids [69]. The acquisition of some stemness properties was suggested in hybrid cells generated from MSC with gastric cancer cells including increased expression of Oct4, Nanog, Sox2 and Lin28. Moreover, expression of CD44 and CD133 on hybrid cells was elevated compared to parental gastric cancer cells. In addition, gastric cancer fusion cells featured a higher proliferative capacity as compared to the parental cell lines [68].

A variety of important questions concerning tumor hybrid cells remain to be elucidated with respect to the existence, frequency and mechanisms of formation in vivo as well as to the role of these fused cell types in tumorigenesis and initiation of metastasis. At least breast and gastric cancer hybrids provide evidence for enhanced tumorigenic and metastatic properties [68, 69]. Nonetheless, several further questions include cell biological properties, chemosensitivities and -resistance and a possible relationship to cancer stem cells [70].

### Molecular and functional consequences during interaction of MSC with tumor cells

During the multistep procedure of tumorigenesis, intercellular communication of diverse cell types within the TME contributes to the malignancy of primary tumor cells as well as their metastatic ability [71]. In co-culture models of MSC populations together with different kinds of breast and ovarian tumor cells, MSC increase the proliferation of cancer cells [38]. Multiple cellular interactions between MSC and breast cancer cells are accompanied by increased growth of breast cancer both in vitro and in vivo [72] including MSC-mediated induction of CD90 expression in the tumor cells. This transient CD90 expression in breast cancer cells results from different kinds of interactions during co-culture with MSC such as microparticles, notch signaling, GJIC or nanotube formation [36–38]. Of interest, MSC functionality can alter during chemotherapy displaying different effects on breast cancer cells [73]. Whereas MSC exhibit close vicinity to breast cancer cells within the tumor microenvironment, there are also cellular interactions observed between MSC and normal human mammary epithelial cells (HMEC) which are usually localized in the normal fibroglandular breast tissue [38].

In ovarian cancer cells, MSC can induce further surface markers besides expression of CD90 such as functional CD73 and CD105. Thus, primary small cell hypercalcemic ovarian carcinoma cells (SCCOHT-1) acquired the capability to metabolize cyclic adenosine 3’, 5’-monophosphate (cAMP) underlining the bidirectional molecular exchange between tumor cells and MSC [38].

Moreover, up-regulated mitotic spindle-associated factors (MZT2A) and epithelial mitogens (EPGN) suggested the promotion of proliferative capacity in ovarian cancer cells. In parallel, a down-modulation of transcription factors like TAL1, transcripts of the basic helix-loop-helix family FOS and FOSB, HES1 and HES5 are also related to the promotion of cancer development. In addition, growth factors of the bone morphogenetic protein family were acquired by ovarian cancer cells in the presence of MSC [38], whereby overactivation of BMP signaling contributes to the development of certain adenocarcinoma. Together, gene inductions during MSC and cancer cell co-culture demonstrated that MSC not only support ovarian cancer cell proliferation capacity but also induce pro-metastatic properties of ovarian cancer cells [74]. Vice versa by looking at MSC, mutual functional alteration during co-culture with tumor cells also include a variety of different gene transcripts which are up- or down-regulated in these stroma/stem cells as analyzed by RNA microarrays [38, 74]. Of interest, MSC expressed increasing levels of epithelial cell-specific transcripts including a group of KRT family genes, which are involved in the production of keratins for supporting the structural framework requirements of epithelial cells. Also certain cell-to-cell interaction related genes, intercellular junction *DSP* gene, the cell-cell adhesion-related genes *MPZL2* and *SCEL*, and the calcium-dependent cell-cell adhesion glycoprotein gene *CDH1* are all up-regulated in MSC after co-culture with ovarian cancer cells. Conversely, a variety of cytokine mRNAs such as CSF3, IL1A, CCL20, LIF, TNF, CXCL1, CXCL2, CXCL3, and CXCL12 are down-regulated in MSC in the presence of ovarian cancer cells [38].

Furthermore, undetectable expression of the epithelial cell adhesion molecule (EpCAM) in normal MSC significantly increased both, at the mRNA and protein level after co-culture with different ovarian cancer cell lines such as SK-OV-3 or NIH:OVCAR-3 [38]. Together these findings substantiate that MSC gain certain epithelial-like cell functionalities during interaction with ovarian cancer cells and may therefore develop an aberrant and more tumor-associated phenotype. Therefore, the mutual bidirectional interactions further suggest a more epithelial-type conversion of MSC compared to transitional properties of mesenchymal characteristics in the ovarian cancer cells.

### MSC and cancer stem cells

Previous work presented evidence for tumor-initiating cells (TIC) in mammary carcinoma also termed cancer stem cells (CSC) with increased expression of mesenchymal characteristics including vimentin, fibronectin and N-cadherin instead of E-cadherin [75]. Moreover, low expression of GPI-anchored sialoglycoprotein cell adhesion molecule CD24 paralleled by high expression of the hyaluronan receptor CD44 as well as expression of aldehyde dehydrogenase 1 are attributed with CSC. Further studies in mammary tumors revealed that IL6 produced by cancer cells interacts with IL6 receptor on aldehyde dehydrogenase 1-positive mesenchymal cells, whereby this IL6 signaling-mediated chemotaxis may facilitate recruitment of further MSC to the tumor microenvironment and induction of CXCL7 production by these cells. Vice versa, MSC-derived CXCL7 stimulates the cancer cells via activation of the CXCR2 receptor and induces the synthesis of additional cytokines such as IL6 and IL8 to generate a positive feedback loop which contributes to increased MSC attraction and enhanced interactions with tumor cells [76].

Following continuous mutual interaction within the TME, cytokines and particularly IL1 released by tumor cells can stimulate arachidonic acid metabolism and subsequent PGE2 production in MSC. Vice versa, released cytokines and PGE2 together can induce β-catenin signaling in the neoplastic cells which contributes to the development of more immature stem cell-like properties [41].

During these interactions characteristics of a mesenchymal phenotype are progressively acquired by the cancer cells [36–38] which may include a MSC-mediated epithelial-to-mesenchymal transition (EMT) in the cancer cells. This suggests a retrodifferentiation process of cancer cells into a stem cell–like phenotype [77, 78] which involves a potential carcinoma stem cell niche (CSCN) [41] provided by interaction of MSC with cancer cells. A corresponding niche-forming property has been assigned to MSC by displaying the capacity to organize the hematopoietic stem cell niche [79, 80]. Alternatively, during cellular interactions or reprogramming MSC can acquire functional properties from the cancer cells which are displayed in an altered tumor-associated mesenchymal stem cell phenotype. Such oncogenic reprogramming can transform MSC into aggressive sarcoma cells [81] and may also play a role in tumors with mesenchymal characteristics such as desmoid tumor [82].

Consequently, the newly arising cancer cell populations after EMT and interaction display an enhanced phenotypic plasticity including metastatic potential and altered responsiveness/resistance to therapeutic approaches.

### MSC interactions and epithelial-mesenchymal transition (EMT)

Conversion of epithelial cells into a mesenchymal phenotype termed as EMT is a prerequisite in physiological processes during early stages of embryonic development including gastrulation and myogenesis [83]. However, EMT is also involved in pathological events such as fibrosis, tumor development and metastasis whereby TGF-β signaling plays an important role [84–86]. EMT as a biological process allows an epithelial cell to undergo complex multiple changes of its cell pattern and morphology which leads to a mesenchymal cell phenotype. This transition is generally delineated by changes of epithelial-like cell properties, for instance 1) down-modulation of E-cadherin for loss apico-basal polarity and cell-cell adhesion, 2) secretion of enzymes such as matrix metalloproteinases to degrade the ECM, and 3) upregulation of mesenchymal markers, e.g. vimentin, N-cadherin and fibronectin, all of which are paralleled by elevated migration, acquired invasiveness and increased resistance to apoptosis [83, 85, 87].

EMT has been implicated in several tumors including breast, ovarian and colon cancer [88–91]. In previous studies, a plethora of oncogenic EMT inducers have been characterized which comprise the EMT transcriptions factors (EMT-TF) Snail1/2, Slug, Twist1 and Zeb1/2 and signaling pathways such as TGFβ and Wnt [92]. Besides the overall common function of the EMT-TF in repressing E-cadherin for loss of cell adhesion, Snail and Slug also regulate tight junction stability and protease expression while Twist1 induces mesenchymal gene expression [83, 93].

Recently, it has been demonstrated that colon cancer cells increased expression of EMT-TF such as Zeb1/2, Slug, Snail and Twist which was paralleled by a downregulation of E-cadherin expression. Moreover, these colon cancer cells acquired the expression of stemness genes including Oct4 and Sox2 after co-culture with adipose tissue-derived MSC. Additionally, the morphology of colon cancer cells was altered to an elongated, fibroblast-like cell shape underlying the conversion to a mesenchymal phenotype. Vice versa, colon cancer cells were able to induce secretion of cytokines (TNFα, IL10, IFNγ) and metastasis-related factors (VEGFC, MMPs) in MSC via activation of Wnt signaling which in turn resulted in activation of Wnt pathways in colon cancer cells. Of interest, inhibition of Wnt signaling reduced the invasiveness and tumorigenicity of cancer cells both in vitro and in vivo [94].

Besides these direct interactions between MSC and colon cancer cells leading to induction of EMT and higher invasiveness, indirect interactions also resulted in EMT induction. Adipose tissue-derived MSC can alter cell confluence and migration of SKBR3 breast cancer cells, increase mammosphere formation, induce EMT, and alter tumor cell morphology [95]. These features were attributed to molecular changes induced by MSC-secreted cytokines and chemokines in breast cancer cells.

Nasopharyngeal carcinoma (NPC) cells exhibited a higher proliferation and migration capacity after uptake of MSC-derived exosomes. Moreover, EMT markers were significantly altered after uptake of exosomes including down-modulation of E-cadherin and upregulation of vimentin and N-cadherin. This indirect communication between MSC-derived exosomes and cancer cells induced EMT, promoted tumor growth in vitro and in vivo and metastasis [96].

Further studies substantiate the occurrence of direct and indirect MSC-cancer cell interactions leading to induction of EMT, thereby altering the cell pattern and morphology of cancer cells to a mesenchymal phenotype which facilitates metastasis to distant tumor sites [38, 41, 97–99].

## Conclusions

Different types of intercellular communication both, indirect and/or direct between MSC and tumor cells (from solid cancers of the breast, ovar, colon, etc.) involve mutual functional alterations whereby the tumor cells acquire certain mesenchymal properties. Depending on the type and the extent of cellular interaction, even completely new tumor cell populations can be formed in the rare event of a MSC and tumor cell fusion. In addition, MSC which are recruited to the invasive tumor sites to initiate regenerative potential are progressively altered into an aberrant MSC phenotype to functionally support tumor cell survival. Therefore, tumor-associated aberrant MSC are involved in tumor cell protection and consequently, contribute to certain effects of chemotherapeutic resistance either directly by expression of protective extracellular matrix proteins as a drug barrier and/or indirectly by promoting EMT of tumor cells and participating in a carcinoma stem cell niche. Such development includes the interplay with various other tumor-associated cell populations and restructure of the ECM, furthermore highlighting the tumor microenvironment as potential therapeutic anti-tumor target.

## Abbreviations

CAF: Carcinoma-associated fibroblast
cAMP: Cyclic adenosine monophosphate
CCL5: CC-chemokine ligand 5
CSC: Cancer stem cells
CSCN: Cancer stem cell niche
DAPT: N-[N-(3,5-difluorophenacetyl-lalanyl)]-S-phenylglycine t-butyl ester
ECM: Extracellular matrix
EMT: Epithelial-mesenchymal transition
EMT-TF: EMT transcription factors
GJIC: Gap junctional intercellular communication
HMEC: Human mammary epithelial cells
IDO: Indoleamine 2,3-dioxygenase
miRs: microRNAs
MSC: Mesenchymal stroma/stem cells
OXPHOS: oxidative phosphorylation
PKA: protein kinase A
RANTES: regulated on activation, normal T cell expressed and secreted
SCCOHT-1: primary small cell hypercalcemic ovarian carcinoma cells
TIC: tumor-initiating cells
TME: tumor microenvironment

## References

[1] Bissell MJ, Hines WC (2011). Why don’t we get more cancer? A proposed role of the microenvironment in restraining cancer progression. Nat Med.

[2] Bissell MJ, Radisky D (2001). Putting tumours in context. Nat Rev Cancer.

[3] Balkwill FR, Capasso M, Hagemann T (2012). The tumor microenvironment at a glance. J Cell Sci.

[4] Whiteside TL (2008). The tumor microenvironment and its role in promoting tumor growth. Oncogene.

[5] Fridman WH, Pages F, Sautes-Fridman C, Galon J (2012). The immune contexture in human tumours: impact on clinical outcome. Nat Rev Cancer.

[6] Coronella JA, Telleman P, Kingsbury GA, Truong TD, Hays S, Junghans RP (2001). Evidence for an antigen-driven humoral immune response in medullary ductal breast cancer. Cancer Res.

[7] Qin Z, Richter G, Schuler T, Ibe S, Cao X, Blankenstein T (1998). B cells inhibit induction of T cell-dependent tumor immunity. Nat Med.

[8] Ishigami S, Natsugoe S, Tokuda K, Nakajo A, Che X, Iwashige H, Aridome K, Hokita S, Aikou T (2000). Prognostic value of intratumoral natural killer cells in gastric carcinoma. Cancer.

[9] Zamarron BF, Chen W (2011). Dual roles of immune cells and their factors in cancer development and progression. Int J Biol Sci.

[10] Hanahan D, Coussens LM (2012). Accessories to the crime: functions of cells recruited to the tumor microenvironment. Cancer Cell.

[11] Armulik A, Genove G, Betsholtz C (2011). Pericytes: developmental, physiological, and pathological perspectives, problems, and promises. Dev Cell.

[12] Cooke VG, LeBleu VS, Keskin D, Khan Z, O’Connell JT, Teng Y, Duncan MB, Xie L, Maeda G, Vong S, Sugimoto H, Rocha RM, Damascena A, Brentani RR, Kalluri R (2012). Pericyte depletion results in hypoxia-associated epithelial-to-mesenchymal transition and metastasis mediated by met signaling pathway. Cancer Cell.

[13] Klopp AH, Gupta A, Spaeth E, Andreeff M, Marini F (2011). Concise review: dissecting a discrepancy in the literature: do mesenchymal stem cells support or suppress tumor growth?. Stem Cells.

[14] Hass R, Otte A (2012). Mesenchymal stem cells as all-round supporters in a normal and neoplastic microenvironment. Cell Commun Signal.

[15] Rhee KJ, Lee JI, Eom YW (2015). Mesenchymal stem cell-mediated effects of tumor support or suppression. Int J Mol Sci.

[16] Barcellos-de-Souza, P., G. Comito, C. Pons-Segura, M.L. Taddei, V. Gori, V. Becherucci, F. Bambi, F. Margheri, A. Laurenzana, M. Del Rosso, P. Chiarugi. Mesenchymal Stem Cells are Recruited and Activated into Carcinoma-Associated Fibroblasts by Prostate Cancer Microenvironment-Derived TGF-beta1. Stem Cells, 2016. doi: 10.1002/stem.2412. 10.1002/stem.2412 27300750

[17] Gilkes DM, Semenza GL, Wirtz D (2014). Hypoxia and the extracellular matrix: drivers of tumour metastasis. Nat Rev Cancer.

[18] Hui L, Chen Y (2015). Tumor microenvironment: sanctuary of the devil. Cancer Lett.

[19] Provenzano PP, Inman DR, Eliceiri KW, Knittel JG, Yan L, Rueden CT, White JG, Keely PJ (2008). Collagen density promotes mammary tumor initiation and progression. BMC Med.

[20] Levental KR, Yu H, Kass L, Lakins JN, Egeblad M, Erler JT, Fong SF, Csiszar K, Giaccia A, Weninger W, Yamauchi M, Gasser DL, Weaver VM (2009). Matrix crosslinking forces tumor progression by enhancing integrin signaling. Cell.

[21] Hass R, Kasper C, Bohm S, Jacobs R (2011). Different populations and sources of human mesenchymal stem cells (MSC): a comparison of adult and neonatal tissue-derived MSC. Cell Commun Signal.

[22] Caplan AI (2009). Why are MSCs therapeutic? New data: new insight. J Pathol.

[23] Pittenger MF, Mackay AM, Beck SC, Jaiswal RK, Douglas R, Mosca JD, Moorman MA, Simonetti DW, Craig S, Marshak DR (1999). Multilineage potential of adult human mesenchymal stem cells. Science.

[24] Bianco P (2014). “Mesenchymal” stem cells. Annu Rev Cell Dev Biol.

[25] Dominici M, Le Blanc K, Mueller I, Slaper-Cortenbach I, Marini F, Krause D, Deans R, Keating A, Prockop D, Horwitz E (2006). Minimal criteria for defining multipotent mesenchymal stromal cells. The international society for cellular therapy position statement. Cytotherapy.

[26] Hoffmann, A., T. Floerkemeier, C. Melzer, R. Hass. Comparison of in vitro-cultivation of human mesenchymal stroma/stem cells derived from bone marrow and umbilical cord. J Tissue Eng Regen Med. 2016. doi:10.1002/term.2153. 10.1002/term.2153 27125777

[27] Otte A, Bucan V, Reimers K, Hass R (2013). Mesenchymal stem cells maintain long-term in vitro stemness during explant culture. Tissue Eng Part C Methods.

[28] Yang Y, Melzer C, Bucan V, von der Ohe J, Otte A, Hass R (2016). Conditioned umbilical cord tissue provides a natural three-dimensional storage compartment as in vitro stem cell niche for human mesenchymal stroma/stem cells. Stem Cell Res Ther.

[29] Majore I, Moretti P, Hass R, Kasper C (2009). Identification of subpopulations in mesenchymal stem cell-like cultures from human umbilical cord. Cell Commun Signal.

[30] Simmons PJ, Torok-Storb B (1991). Identification of stromal cell precursors in human bone marrow by a novel monoclonal antibody, STRO-1. Blood.

[31] Crisan M, Yap S, Casteilla L, Chen CW, Corselli M, Park TS, Andriolo G, Sun B, Zheng B, Zhang L, Norotte C, Teng PN, Traas J, Schugar R, Deasy BM, Badylak S, Buhring HJ, Giacobino JP, Lazzari L, Huard J, Peault B (2008). A perivascular origin for mesenchymal stem cells in multiple human organs. Cell Stem Cell.

[32] Honczarenko M, Le Y, Swierkowski M, Ghiran I, Glodek AM, Silberstein LE (2006). Human bone marrow stromal cells express a distinct set of biologically functional chemokine receptors. Stem Cells.

[33] Kuci S, Kuci Z, Kreyenberg H, Deak E, Putsch K, Huenecke S, Amara C, Koller S, Rettinger E, Grez M, Koehl U, Latifi-Pupovci H, Henschler R, Tonn T, von Laer D, Klingebiel T, Bader P (2010). CD271 antigen defines a subset of multipotent stromal cells with immunosuppressive and lymphohematopoietic engraftment-promoting properties. Haematologica.

[34] Kuroda Y, Kitada M, Wakao S, Nishikawa K, Tanimura Y, Makinoshima H, Goda M, Akashi H, Inutsuka A, Niwa A, Shigemoto T, Nabeshima Y, Nakahata T, Nabeshima Y, Fujiyoshi Y, Dezawa M (2010). Unique multipotent cells in adult human mesenchymal cell populations. Proc Natl Acad Sci U S A.

[35] Karnoub AE, Dash AB, Vo AP, Sullivan A, Brooks MW, Bell GW, Richardson AL, Polyak K, Tubo R, Weinberg RA (2007). Mesenchymal stem cells within tumour stroma promote breast cancer metastasis. Nature.

[36] Mandel K, Yang Y, Schambach A, Glage S, Otte A, Hass R (2013). Mesenchymal stem cells directly interact with breast cancer cells and promote tumor cell growth in vitro and in vivo. Stem Cells Dev.

[37] Yang Y, Bucan V, Baehre H, von der Ohe J, Otte A, Hass R (2015). Acquisition of new tumor cell properties by MSC-derived exosomes. Int J Oncol.

[38] Yang Y, Otte A, Hass R (2015). Human mesenchymal stroma/stem cells exchange membrane proteins and alter functionality during interaction with different tumor cell lines. Stem Cells Dev.

[39] Caplan AI, Correa D (2011). The MSC: an injury drugstore. Cell Stem Cell.

[40] Chiarugi P, P. Cirri. Metabolic exchanges within tumor microenvironment. Cancer Lett. 2016;380:272–80. 10.1016/j.canlet.2015.10.027 26546872

[41] Li HJ, Reinhardt F, Herschman HR, Weinberg RA (2012). Cancer-stimulated mesenchymal stem cells create a carcinoma stem cell niche via prostaglandin E2 signaling. Cancer Discov.

[42] Yuan Y, Lu X, Tao CL, Chen X, Shao HW, Huang SL (2013). Forced expression of indoleamine-2,3-dioxygenase in human umbilical cord-derived mesenchymal stem cells abolishes their anti-apoptotic effect on leukemia cell lines in vitro. In Vitro Cell Dev Biol Anim.

[43] Naderi EH, Findley HW, Ruud E, Blomhoff HK, Naderi S (2009). Activation of cAMP signaling inhibits DNA damage-induced apoptosis in BCP-ALL cells through abrogation of p53 accumulation. Blood.

[44] Naderi EH, Jochemsen AG, Blomhoff HK, Naderi S (2011). Activation of cAMP signaling interferes with stress-induced p53 accumulation in ALL-derived cells by promoting the interaction between p53 and HDM2. Neoplasia.

[45] Naderi EH, Skah S, Ugland H, Myklebost O, Sandnes DL, Torgersen ML, Josefsen D, Ruud E, Naderi S, Blomhoff HK (2015). Bone marrow stroma-derived PGE2 protects BCP-ALL cells from DNA damage-induced p53 accumulation and cell death. Mol Cancer.

[46] Otte A, Rauprich F, von der Ohe J, Hillemanns P, Hass R (2014). Interference of Ca(2)(+) with the proliferation of SCCOHT-1 and ovarian adenocarcinoma cells. Int J Oncol.

[47] Bonuccelli G, Avnet S, Grisendi G, Salerno M, Granchi D, Dominici M, Kusuzaki K, Baldini N (2014). Role of mesenchymal stem cells in osteosarcoma and metabolic reprogramming of tumor cells. Oncotarget.

[48] Peppicelli S, Bianchini F, Calorini L (2014). Extracellular acidity, a “reappreciated” trait of tumor environment driving malignancy: perspectives in diagnosis and therapy. Cancer Metastasis Rev.

[49] Lee Y, El Andaloussi S, Wood MJ (2012). Exosomes and microvesicles: extracellular vesicles for genetic information transfer and gene therapy. Hum Mol Genet.

[50] Friedl P, Alexander S (2011). Cancer invasion and the microenvironment: plasticity and reciprocity. Cell.

[51] Vallabhaneni KC, Penfornis P, Dhule S, Guillonneau F, Adams KV, Mo YY, Xu R, Liu Y, Watabe K, Vemuri MC, Pochampally R (2015). Extracellular vesicles from bone marrow mesenchymal stem/stromal cells transport tumor regulatory microRNA, proteins, and metabolites. Oncotarget.

[52] Chowdhury R, Webber JP, Gurney M, Mason MD, Tabi Z, Clayton A (2015). Cancer exosomes trigger mesenchymal stem cell differentiation into pro-angiogenic and pro-invasive myofibroblasts. Oncotarget.

[53] Wu S, Ju GQ, Du T, Zhu YJ, Liu GH (2013). Microvesicles derived from human umbilical cord Wharton’s jelly mesenchymal stem cells attenuate bladder tumor cell growth in vitro and in vivo. PLoS One.

[54] Del Papa B, Sportoletti P, Cecchini D, Rosati E, Balucani C, Baldoni S, Fettucciari K, Marconi P, Martelli MF, Falzetti F, Di Ianni M (2013). Notch1 modulates mesenchymal stem cells mediated regulatory T-cell induction. Eur J Immunol.

[55] Kopan R, Ilagan MX (2009). The canonical Notch signaling pathway: unfolding the activation mechanism. Cell.

[56] Geling A, Steiner H, Willem M, Bally-Cuif L, Haass C (2002). A gamma-secretase inhibitor blocks Notch signaling in vivo and causes a severe neurogenic phenotype in zebrafish. EMBO Rep.

[57] Kabashima-Niibe A, Higuchi H, Takaishi H, Masugi Y, Matsuzaki Y, Mabuchi Y, Funakoshi S, Adachi M, Hamamoto Y, Kawachi S, Aiura K, Kitagawa Y, Sakamoto M, Hibi T (2013). Mesenchymal stem cells regulate epithelial-mesenchymal transition and tumor progression of pancreatic cancer cells. Cancer Sci.

[58] Kandouz M, Batist G (2010). Gap junctions and connexins as therapeutic targets in cancer. Expert Opin Ther Targets.

[59] Gurke S, Barroso JF, Gerdes HH (2008). The art of cellular communication: tunneling nanotubes bridge the divide. Histochem Cell Biol.

[60] Levchenko A, Mehta BM, Niu X, Kang G, Villafania L, Way D, Polycarpe D, Sadelain M, Larson SM (2005). Intercellular transfer of P-glycoprotein mediates acquired multidrug resistance in tumor cells. Proc Natl Acad Sci U S A.

[61] Caicedo A, Fritz V, Brondello JM, Ayala M, Dennemont I, Abdellaoui N, de Fraipont F, Moisan A, Prouteau CA, Boukhaddaoui H, Jorgensen C, Vignais ML (2015). MitoCeption as a new tool to assess the effects of mesenchymal stem/stromal cell mitochondria on cancer cell metabolism and function. Sci Rep.

[62] Joly E, Hudrisier D (2003). What is trogocytosis and what is its purpose?. Nat Immunol.

[63] Rafii A, Mirshahi P, Poupot M, Faussat AM, Simon A, Ducros E, Mery E, Couderc B, Lis R, Capdet J, Bergalet J, Querleu D, Dagonnet F, Fournie JJ, Marie JP, Pujade-Lauraine E, Favre G, Soria J, Mirshahi M (2008). Oncologic trogocytosis of an original stromal cells induces chemoresistance of ovarian tumours. PLoS One.

[64] Ogle BM, Cascalho M, Platt JL (2005). Biological implications of cell fusion. Nat Rev Mol Cell Biol.

[65] Tosun S, Fried S, Niggemann B, Zanker KS, Dittmar T. Hybrid Cells Derived from Human Breast Cancer Cells and Human Breast Epithelial Cells Exhibit Differential TLR4 and TLR9 Signaling. Int J Mol Sci. 2016;17(5). 10.3390/ijms17050726 PMC4881548 27187369

[66] Noubissi FK, Harkness T, Alexander CM, Ogle BM (2015). Apoptosis-induced cancer cell fusion: a mechanism of breast cancer metastasis. FASEB J.

[67] Wei HJ, Nickoloff JA, Chen WH, Liu HY, Lo WC, Chang YT, Yang PC, Wu CW, Williams DF, Gelovani JG, Deng WP (2014). FOXF1 mediates mesenchymal stem cell fusion-induced reprogramming of lung cancer cells. Oncotarget.

[68] Xue J, Zhu Y, Sun Z, Ji R, Zhang X, Xu W, Yuan X, Zhang B, Yan Y, Yin L, Xu H, Zhang L, Zhu W, Qian H (2015). Tumorigenic hybrids between mesenchymal stem cells and gastric cancer cells enhanced cancer proliferation, migration and stemness. BMC Cancer.

[69] Rappa G, Mercapide J, Lorico A (2012). Spontaneous formation of tumorigenic hybrids between breast cancer and multipotent stromal cells is a source of tumor heterogeneity. Am J Pathol.

[70] Dittmar T, Nagler C, Niggemann B, Zanker KS (2013). The dark side of stem cells: triggering cancer progression by cell fusion. Curr Mol Med.

[71] Ungefroren H, Sebens S, Seidl D, Lehnert H, Hass R (2011). Interaction of tumor cells with the microenvironment. Cell Commun Signal.

[72] Muehlberg FL, Song YH, Krohn A, Pinilla SP, Droll LH, Leng X, Seidensticker M, Ricke J, Altman AM, Devarajan E, Liu W, Arlinghaus RB, Alt EU (2009). Tissue-resident stem cells promote breast cancer growth and metastasis. Carcinogenesis.

[73] Skolekova S, Matuskova M, Bohac M, Toro L, Durinikova E, Tyciakova S, Demkova L, Gursky J, Kucerova L (2016). Cisplatin-induced mesenchymal stromal cells-mediated mechanism contributing to decreased antitumor effect in breast cancer cells. Cell Commun Signal.

[74] Lis R, Touboul C, Halabi NM, Madduri AS, Querleu D, Mezey J, Malek JA, Suhre K, Rafii A (2014). Mesenchymal cell interaction with ovarian cancer cells induces a background dependent pro-metastatic transcriptomic profile. J Transl Med.

[75] Mani SA, Guo W, Liao MJ, Eaton EN, Ayyanan A, Zhou AY, Brooks M, Reinhard F, Zhang CC, Shipitsin M, Campbell LL, Polyak K, Brisken C, Yang J, Weinberg RA (2008). The epithelial-mesenchymal transition generates cells with properties of stem cells. Cell.

[76] Liu S, Ginestier C, Ou SJ, Clouthier SG, Patel SH, Monville F, Korkaya H, Heath A, Dutcher J, Kleer CG, Jung Y, Dontu G, Taichman R, Wicha MS (2011). Breast cancer stem cells are regulated by mesenchymal stem cells through cytokine networks. Cancer Res.

[77] Hass R (2009). Retrodifferentiation--a mechanism for cellular regeneration?. Biol Chem.

[78] Hass R, Gunji H, Datta R, Kharbanda S, Hartmann A, Weichselbaum R, Kufe D (1992). Differentiation and retrodifferentiation of human myeloid leukemia cells is associated with reversible induction of cell cycle-regulatory genes. Cancer Res.

[79] Bianco P (2011). Bone and the hematopoietic niche: a tale of two stem cells. Blood.

[80] Friedenstein AJ, Latzinik NW, Grosheva AG, Gorskaya UF (1982). Marrow microenvironment transfer by heterotopic transplantation of freshly isolated and cultured cells in porous sponges. Exp Hematol.

[81] Eid JE, Garcia CB (2015). Reprogramming of mesenchymal stem cells by oncogenes. Semin Cancer Biol.

[82] Wu C, Amini-Nik S, Nadesan P, Stanford WL, Alman BA (2010). Aggressive fibromatosis (desmoid tumor) is derived from mesenchymal progenitor cells. Cancer Res.

[83] Micalizzi DS, Farabaugh SM, Ford HL (2010). Epithelial-mesenchymal transition in cancer: parallels between normal development and tumor progression. J Mammary Gland Biol Neoplasia.

[84] Humphreys BD, Lin SL, Kobayashi A, Hudson TE, Nowlin BT, Bonventre JV, Valerius MT, McMahon AP, Duffield JS (2010). Fate tracing reveals the pericyte and not epithelial origin of myofibroblasts in kidney fibrosis. Am J Pathol.

[85] Smith B.N, N.A. Bhowmick. Role of EMT in Metastasis and Therapy Resistance. J Clin Med, 2016. 5(2). 10.3390/jcm5020017 PMC4773773 26828526

[86] Laurenzana A, Biagioni A, Bianchini F, Peppicelli S, Chilla A, Margheri F, Luciani C, Pimpinelli N, Del Rosso M, Calorini L, Fibbi G (2015). Inhibition of uPAR-TGFbeta crosstalk blocks MSC-dependent EMT in melanoma cells. J Mol Med (Berl).

[87] Kalluri R, Weinberg RA (2009). The basics of epithelial-mesenchymal transition. J Clin Invest.

[88] Brabletz T, Hlubek F, Spaderna S, Schmalhofer O, Hiendlmeyer E, Jung A, Kirchner T (2005). Invasion and metastasis in colorectal cancer: epithelial-mesenchymal transition, mesenchymal-epithelial transition, stem cells and beta-catenin. Cells Tissues Organs.

[89] Trimboli AJ, Fukino K, de Bruin A, Wei G, Shen L, Tanner SM, Creasap N, Rosol TJ, Robinson ML, Eng C, Ostrowski MC, Leone G (2008). Direct evidence for epithelial-mesenchymal transitions in breast cancer. Cancer Res.

[90] Vergara D, Merlot B, Lucot JP, Collinet P, Vinatier D, Fournier I, Salzet M (2010). Epithelial-mesenchymal transition in ovarian cancer. Cancer Lett.

[91] Chaturvedi S, Hass R (2011). Extracellular signals in young and aging breast epithelial cells and possible connections to age-associated breast cancer development. Mech Ageing Dev.

[92] Lamouille S, Xu J, Derynck R (2014). Molecular mechanisms of epithelial-mesenchymal transition. Nat Rev Mol Cell Biol.

[93] Lv N, Shan Z, Gao Y, Guan H, Fan C, Wang H, Teng W (2016). Twist1 regulates the epithelial-mesenchymal transition via the NF-kappaB pathway in papillary thyroid carcinoma. Endocrine.

[94] Chen D, Liu S, Ma H, Liang X, Ma H, Yan X, Yang B, Wei J, Liu X (2015). Paracrine factors from adipose-mesenchymal stem cells enhance metastatic capacity through Wnt signaling pathway in a colon cancer cell co-culture model. Cancer Cell Int.

[95] Kucerova L, Skolekova S, Matuskova M, Bohac M, Kozovska Z (2013). Altered features and increased chemosensitivity of human breast cancer cells mediated by adipose tissue-derived mesenchymal stromal cells. BMC Cancer.

[96] Shi S, Zhang Q, Xia Y, You B, Shan Y, Bao L, Li L, You Y, Gu Z (2016). Mesenchymal stem cell-derived exosomes facilitate nasopharyngeal carcinoma progression. Am J Cancer Res.

[97] Li T, Zhang C, Ding Y, Zhai W, Liu K, Bu F, Tu T, Sun L, Zhu W, Zhou F, Qi W, Hu J, Chen H, Sun X (2015). Umbilical cord-derived mesenchymal stem cells promote proliferation and migration in MCF-7 and MDA-MB-231 breast cancer cells through activation of the ERK pathway. Oncol Rep.

[98] Martin FT, Dwyer RM, Kelly J, Khan S, Murphy JM, Curran C, Miller N, Hennessy E, Dockery P, Barry FP, O’Brien T, Kerin MJ (2010). Potential role of mesenchymal stem cells (MSCs) in the breast tumour microenvironment: stimulation of epithelial to mesenchymal transition (EMT). Breast Cancer Res Treat.

[99] So KA, Min KJ, Hong JH, Lee JK (2015). Interleukin-6 expression by interactions between gynecologic cancer cells and human mesenchymal stem cells promotes epithelial-mesenchymal transition. Int J Oncol.

